# Supplementary material for: Ketamine Improves Desensitization of µ-Opioid Receptors Induced by Repeated Treatment with Fentanyl but Not with Morphine
Source: Biomolecules. 2022 Mar 10;12(3):426. doi: 10.3390/biom12030426 (PMC8946650; doi:10.3390/biom12030426)
Supplement: Supplementary file 1 [file biomolecules-12-00426-s001.zip › biomolecules-1616356-supplementary.pdf]

Supplementary Material

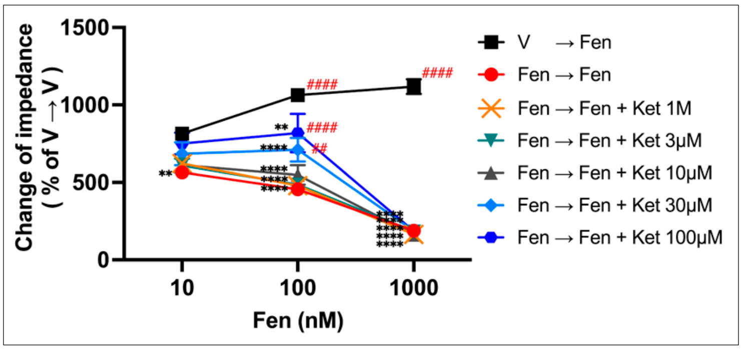


Figure S1: Two-way ANOVA followed by the post hoc Tukey's test for fentanyl dose by ketamine dose in MOR-expressing cells using the CellKeyTM assay.

A two-way ANOVA revealed significant effects of fentanyl dose ($F (2, 105) = 122.6$, $p < 0.0001$, partial $\eta^2$ ($\eta_p^2$) = 0.700), ketamine dose ($F (6, 105) = 58.9$, $p < 0.0001$, $\eta_p^2 = 0.770$) and interaction ($F (12, 105) = 11.3$, $p < 0.0001$, $\eta_p^2 = 0.563$). All data are presented as means ± standard error of mean (SEM) (n = 6-12). $^{**}P < 0.01$, $^{****}P < 0.0001$ in comparison to vehicle to fentanyl group; $^{\#\#}P < 0.01$, $^{\#\#\#\#}P < 0.0001$ in comparison to repeated fentanyl without ketamine pretreatment; ns, not significant; V, vehicle; Fen, fentanyl; Ket, ketamine.

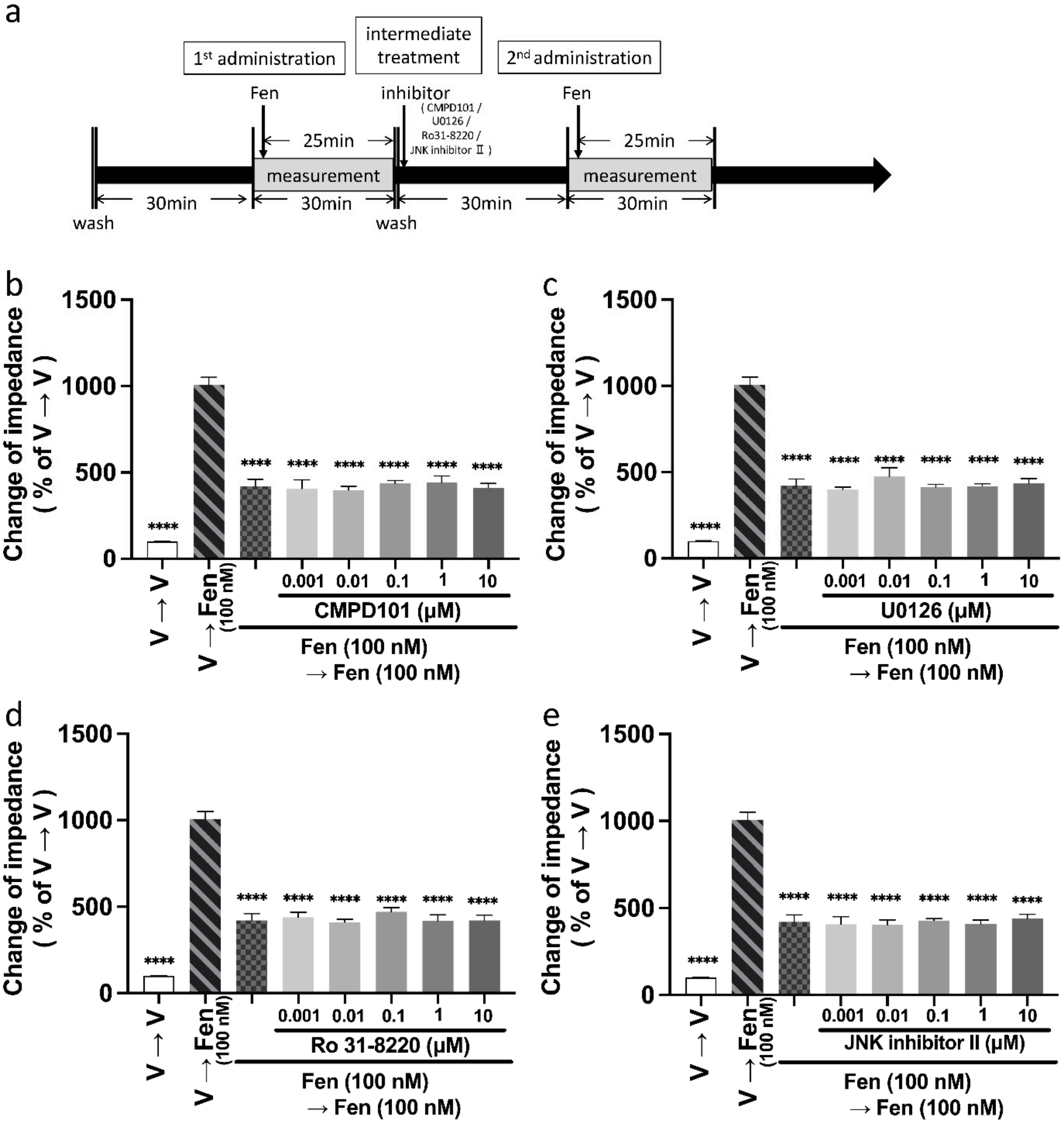


Figure S2: Effects of intermediate treatment with intracellular signal inhibitors on ketamine-induced decrease in MOR activity caused by repeated administration of fentanyl in MOR-expressing cells using the CellKey™ assay.

Each intracellular signal inhibitor was administered for 30 min before the second administration of 100 nM fentanyl (a). Effects of intermediate treatment with CMPD101 (b), U0126 (c), Ro 31-8220 (d) and JNK inhibitor II (e) at concentrations of 0.001–10 μM in the absence of ketamine on ketamine-induced decrease in MOR activity caused by repeated administration of 100 nM fentanyl (one-way ANOVA followed by the post-hoc Tukey's test in comparison to the vehicle to fentanyl group). All data are presented as means ± SEM (n = 6-12). ns, not significant; V, vehicle; Fen, 100 nM fentanyl.

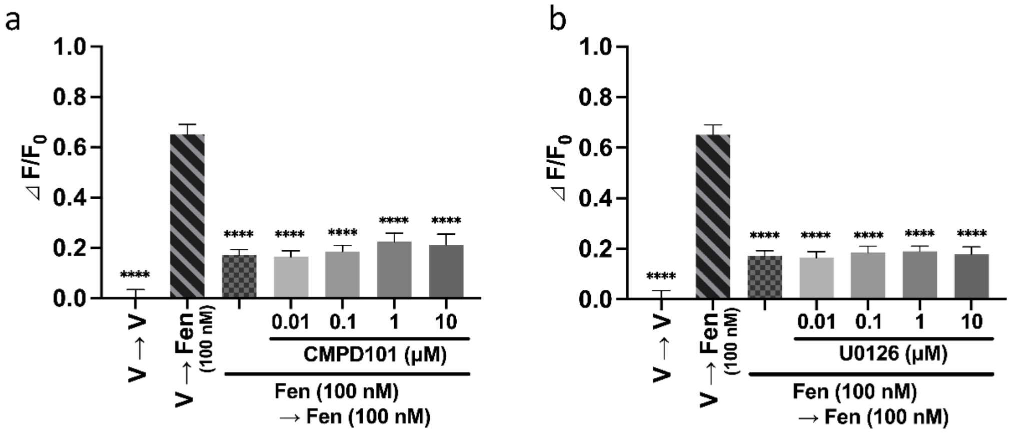


Figure S3: Effects of intermediate treatment with intracellular signal inhibitors on the rescue of intracellular cAMP induced by repeated administration of opioids in MOR-expressing cells using the cADDis cAMP assay.

Effects of 0.01–10 μM of CMPD101 (a) and U0126 (b) on the rescue of intracellular cAMP induced by repeated administration of 100 nM fentanyl in the absence of ketamine (one-way ANOVA followed by the post-hoc Tukey's test in comparison to the vehicle to fentanyl group). All data are presented as means ± SEM (n = 6). ns, not significant; V, vehicle; Fen, 100 nM fentanyl.

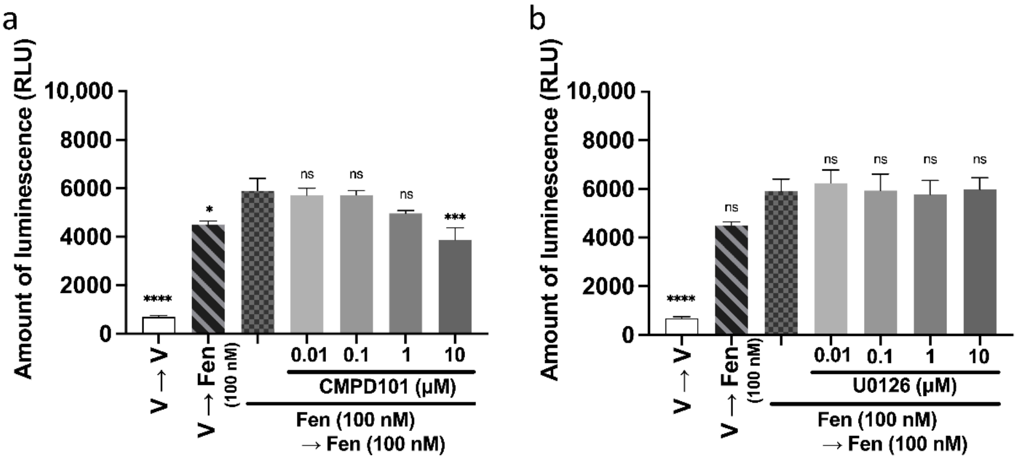


Figure S4: Effects of intermediate treatment with intracellular signal inhibitors on changes in the β-arrestin recruitment levels to MOR induced by repeated administration of opioids in MOR-expressing cells using the PathHunter® eXpress β-arrestin assay.

Effects of 0.01–10 μM of CMPD101 (a) and U0126 (b) on changes in the β-arrestin recruitment levels to MOR induced by repeated administration of 100 nM fentanyl in the absence of ketamine (one-way ANOVA followed by the post-hoc Tukey's test in comparison to the repeated administration of fentanyl group). All data are presented as means ± SEM (n = 6). * $P < 0.05$; *** $P < 0.001$; **** $P < 0.0001$; ns, not significant; V, vehicle; Fen, 100 nM fentanyl.
